# Detection of Amyloid Status and Preclinical Alzheimer's Disease Using Cognivue Clarity, An Adaptive Psychophysics Computerized Cognitive Battery in The Bio‐Hermes Study

**DOI:** 10.1002/alz.086341

**Published:** 2025-01-09

**Authors:** James E Galvin, Michael J Kleiman, Paul W. Estes, Heather M. Harris, Cognivue, Inc.

**Affiliations:** ^1^ Cognivue, Inc., Victor, NY USA; ^2^ University of Miami, Miami, FL USA; ^3^ University of Miami Miller School of Medicine, Boca Raton, FL USA

## Abstract

**Background:**

An easy and reliable method for detection of Alzheimer's Disease (AD) and mild cognitive impairment (MCI) is critical for clinical trial enrollment. In the era of amyloid‐lowering therapies, there is a need to identify individuals likely to have amyloid to enrich recruitment and lower costs related to amyloid PET. In addition, a subset of cognitively normal individuals have amyloid deposition (Preclinical AD) but to date there is no cognitive assessment or screening method that can detect these individuals in the absence of expensive biomarkers. Cognivue Clarity is an adaptive psychophysics computerized cognitive assessment generating a global score and 10 subtest scores.

**Methods:**

As part of the Bio‐Hermes study, sponsored by the Global Alzheimer’s Platform Foundation, Cognivue Clarity was administered to 964 individuals who also had amyloid PET, plasma amyloid and tau measures, ApoE genotyping, MMSE, Functional Activities Questionnaire (FAQ), and Rey Auditory Verbal Learning Task (RAVLT). Cognivue Clarity performance was compared between (1) clinically‐defined, (2) biomarker‐defined, and (3) clinicopathological‐defined groups.

**Results:**

The sample had a mean age of 72.0 ± 6.7y, 15.5 ± 2.7y education, 55.9% females and 23.0% individuals from underrepresented groups. Clinical groups included 42.8% Healthy, 31.6% MCI, and 25.5% Probable AD. Amyloid PET was positive in 62.7%. Clinicopathological groups included 33.5% Healthy, 10.3% Preclinical AD, 27.2% MCI/AD, and 29.1% non‐AD. While Cognivue, MMSE, FAQ, and RAVLT scores were different between impaired vs unimpaired and amyloid positive vs amyloid negative individuals, only Cognivue Clarity was different between Healthy vs Preclinical AD (p=.009). Three subtests [Shape Discrimination (p=.002), Visual Salience (p=.005), Adaptive Motor Control (p=.004)] and their mean (p<.001) differentiated True Controls from Preclinical AD with an area under the curve of 0.634 (95%CI:0.570‐0.698, p<.001) and were moderately correlated with Amyloid PET Centiloid (r=‐.308) and pTau217 (r=‐.315). ApoE carriers had lower scores than non‐carriers (p=.02).

**Conclusions:**

Cognivue Clarity, a 10‐minute computerized cognitive battery, can detect individuals with cognitive impairment and identify individuals likely to have amyloid positivity. Cognivue Clarity is the first test able to identify individuals with Preclinical AD. This has great potential as an enrichment strategy for AD clinical trials testing amyloid‐lowering therapies and AD prevention.

